# Rape Survivors’ Experience of the UK Criminal Justice System: A Qualitative Study

**DOI:** 10.3390/bs16050699

**Published:** 2026-05-04

**Authors:** Hannah Shone, Alison Woodward, Helen Stevens, Peter James Hills

**Affiliations:** 1 Faculty of Science and Technology, Bournemouth University, Fern Barrow, Poole BH12 9BB, UK; 2 Sexual Trauma and Recovery Services, P.O. Box 7697, Poole BH15 9GN, UK; 3 School of Sciences, Bath Spa University, Newton Park, Newton St Loe BA2 9BN, UK

**Keywords:** sexual violence, criminal justice, survivors’ experience, rape, trauma

## Abstract

The conviction rate for sexual assault and rape in the UK has decreased since the 1980s. In part, this has been due to survivors’ negative experiences during their journey through the criminal justice system. While thorough reviews of the criminal justice system have taken place and recommendations have been made, there is a lack of psychological evaluation of the effects of this journey. In this study, we interviewed eight survivors who had gone through the criminal justice system, asking about their experiences and their impact. Thematic analysis, with triangulation, was undertaken, revealing that the negative experiences of feeling let down and the disjointed communication led to prolonged mental health difficulties. The interrelated subthemes highlighted how survivors’ expectations, potentially from seeking justice, were different from reality. Their reality of delayed, poor, and misguided communication from members of the criminal justice system made them feel abandoned, helpless, and powerless. The consequence of this was negative strain, additional trauma, and prolonged distress. These results are interpreted within a framework of the practicalities of investigating serious sexual assault and how best to support survivors of sexual violence while they are treated as witnesses under the criminal justice system in the UK.

## 1. Introduction

The United Kingdom has one of the worst conviction rates for sexual assault in Europe. Research shows a steady decrease in sexual assault convictions from 24% in 1985 to only 7% in 2000 (Hohl & Stanko, 2015) and less than 1% in 2019 (Hohl, 2022). While there is a decrease in convictions, there is a significant increase in the reporting of sexual assault and rape to the police (Home Office, 2019). The startling figures led to an evaluation of the entire criminal justice system and its approach to rape, commissioned by the HM Inspectorate of Constabulary and Fire and Rescue Services (HMICFRS) and HM Crown Prosecution Service Inspectorate (HMCPSI), published in 2021 (HMINCFRS, 2021).

The HMINCFRS (2021) report highlighted how police, investigative, and legal practices throughout the criminal justice system influence the way a survivor of sexual violence feels. In addition, it stated that many of the actions of those working in the criminal justice system occur as a result of poor standards of training and the historical position of the law (where, historically, rape could not occur within marriage, for example). To clarify and expand on these points, it is necessary to briefly and simply explain the criminal justice process in England and Wales^1^.

At some point after an offence occurs (whether after hours, days, or years), the survivor^2^ can choose to report their offence to the police. This can be done face-to-face, over the phone, or using an online submission form. The officer who takes the initial details may not be specially trained in sexual offences. Typically, a specially trained officer will then interview the survivor to collect a statement and evidence to support the police’s investigation. The survivor is likely to need to provide other sources of evidence, including biological samples (for recent offences) and complete access to their phone and other electronic devices. All this information is used by the police to gather evidence to determine if a charge should be made. Once the police investigation is complete, the evidence is submitted to the Crown Prosecution Service (CPS), who determine whether the evidence is sufficient to likely result in a conviction and whether it is in the public interest to pursue a conviction. If taken forward to court, the CPS lawyers will likely discuss the case, evidence, and statement with the survivor on multiple occasions in preparation for court. At court, the survivor is treated as a witness, and their evidence is presented and then cross-examined by the defence attorney. At a minimum, the survivor will need to recount their traumatic experience three times during the process. This retelling of the assault can lead to revictimization and secondary trauma (e.g., Martin & Powell, 1994).

The summary of the criminal justice system provided highlights many reasons why the conviction rate for sexual violence is so low. Indeed, Champion et al. (2021), in their detailed review of the HMINCFRS (2021) report, highlight issues survivors report during each stage of the criminal justice system. Champion et al. (2021) offered several thoughts and reflections that their survivors provided when they reported their assault to the police. As one might expect, experiences were mixed depending on the police officer (and their expertise) and the amount of support offered to survivors. While some reported that police officers were ‘kind, empathetic, and respectful’, others were reported as ‘judgmental, disbelieving, “robotic,” and lacking empathy’ (p. 5). It is well known that negative reactions from members of the criminal justice system can further exacerbate secondary victimization (Campbell & Raja, 1999). Secondary victimization occurs when survivors experience prolonged negative and judgmental attitudes and behaviours that can lead to feelings of alienation, distrust, guilt, depression, anxiety, and post-traumatic stress symptoms (Campbell & Raja, 2005).

Champion et al. (2021) also noted the impact of the time taken for the case to go through the police (and the entire criminal justice system) had on survivors, with little communication or regard for the survivor’s wellbeing. It is known that the average time to go from reporting an assault to the police to a verdict in court has increased to 338 days (Ministry of Justice, 2026). The results of Champion et al. were mirrored in a previous study investigating how US college students felt whilst reporting experiences of sexual violence (Park, 2015). Park found that while there were some who had experienced positive interactions with law enforcement officers, most had very negative and damaging experiences, including victim blaming, being held for investigations, and a lack of communication from the criminal justice system. While Champion et al.’s work is highly useful, it did not address the psychological impact of progressing through the criminal justice system, which is the focus of this work.

The psychological consequence of poor communication and waiting for an outcome or progress after surviving a serious sexual assault cannot be understated. Indeed, it is the focus of much support offered by specialist support services to survivors of sexual violence. Because people can draw inferences about their own status and self-worth from interactions with state representatives (i.e., the police and CPS solicitors, Tyler & Lind, 1992), poor communication will damage self-esteem and violate the survivor’s dignity (Lorenz & Jacobsen, 2024). Prolonged waiting delays recovery, because the survivor knows they will have to relive the trauma when dealing with lawyers and then in court. Such delays can indicate to survivors that they are not a priority (Jordan, 2022), impacting whether they feel believed and how they can move forward. This can cause prolonged re-traumatization, secondary victimization and negatively impacts the survivor’s quality of life (Hohl et al., 2022, 2025). Conversely, support that makes survivors feel emotionally supported, listened to, and understood helps survivors on their journey through the criminal justice system (Horvath et al., 2021; Lovett et al., 2004; Walker et al., 2020) and in their recovery.

Our research was designed to investigate the experiences of survivors of sexual assault and rape who have reported their assault to the police in the UK. While Champion et al. (2021) conducted a thorough review of the issues experienced by survivors of sexual violence in the criminal justice system in the UK, their work stemmed from a legal and criminological perspective. There is a need to conduct similar work from a psychological perspective to establish key themes that drive how future support for survivors could be directed.

## 2. Method

### 2.1. Participants

A purposive sample of eight survivors of sexual violence was recruited from STARS Dorset^3^. The Clinical Lead for STARS Dorset verified who would be suitable for participating in this study based on how far through the service they had got to and whether participating could hinder recovery. These individuals were approached to participate by the charity sending them an invitation to participate with the Participant Information Sheet. All participants who approached opted to take part in the study. Individuals who had agreed to participate were further screened by STARS Dorset to ensure participating in this study would not have any psychological negative impact on them. This involved ensuring that the client was emotionally robust enough to recount their experiences of the criminal justice system.

Participants were aged 18 or over, and all had an experience of sexual assault or rape (either recent or historic), which they had reported to the police within two years prior to the interviews taking place. Five participants had taken their case to court with three guilty verdicts, two not-guilty verdicts, one guilty plea, and one hung jury (for some participants there were multiple offences). Of the remaining participants, one was waiting on whether they would be going to court, and two others did not have their case taken to court through a “no further action” decision by the police (*n* = 1) or CPS (*n* = 1). Participants’ average age was 42 years (range: 21 to 63). All participants defined their gender as female and their ethnicity as White.

The number of participants chosen was to provide sufficient data without over-recruiting for a sensitive topic. While the number of participants is relatively small and geographically restricted, the consistency of the results with previous literature gives confidence in the generalizability of the results beyond that of our participants. Even though we acknowledge our position within social constructivism, we consider the experiences of our participants as not unique. Nonetheless, as with many qualitative studies, this limitation is noted.

### 2.2. Procedure

Interviews were held either online or in-person, based on health constraints (participant mobility or illness) and the participants’ preferences. In-person interviews took place at the offices of STARS Dorset in a private interview room. The in-person interviews were audio-recorded on a portable recording device. Participants who had an online interview were required to use a personal electronic device, such as a laptop or mobile phone, to access Zoom and complete the interview in a quiet, calm space. Interviews conducted online were recorded through Zoom cloud recording. Participants were informed that the interviews would be recorded ahead of time. All interviews were conducted by the first author with the second author in attendance to provide clinical support as required.

At the start of the interviews, participants were presented with the Participant Information Sheet and consent form. Participants were required to read this and confirm they were still happy to participate. Participants with an online interview were required to sign the consent form online and email it back to the researcher before the interview commenced.

At the beginning of the interview, participants were asked to confirm verbally that they were still happy to participate. The interviewer introduced herself to the participants and explained the structure of the interview. Participants were advised they could stop or pause the interview at any time and that support was available. Participants were also reminded that the interview was about their experience of the criminal justice system rather than their experiences of sexual violence. This was done to avoid re-traumatizing the participants. The interviews lasted from 41 to 79 min (mean = 57 mins).

To begin with, participants were asked their age, followed by a broad question asking about their overall experience and feelings of reporting the sexual assault and going through the criminal justice system. This then allowed further questions to be asked to gain more detail on reporting sexual assault or rape to the police and going to court. This way, participants were eased into the interview, allowing them to become more comfortable with the setting, rather than being asked more specific questions too early in the interview. Subsequently, participants were asked a series of questions in a semi-structured interview format. Questions focused on the participants’ experience of reporting the sexual assault to the police, their experience of going through the criminal justice system (if relevant), and then for an overall reflection on how they feel about their experience of going through this process. To aid this reflection, a question about the ‘not proven’ verdict in Scotland was also included (Bray, 2005).

Following the interview, participants received a 10-min debrief session with the Clinical Lead at STARS Dorset. This debrief checked in on how the participant was feeling, confirmed whether any further support needed to be organized for the participant and reiterated the participant’s right to withdraw. A debrief sheet was emailed to the participant, stating the participant’s rights and including key contact details in case further support is needed. No participants withdrew from taking part in this study.

### 2.3. Data Analysis

Once the audio files were transcribed verbatim (and checked), the original audio files were deleted. Participants were given pseudonyms, and their real names were not used during the analysis. Any personally identifiable information present in the interviews was removed in the transcription. Data was analyzed using Thematic Analysis (Braun & Clarke, 2006). This method of analysis was chosen because no pre-existing theory was generated about sexual assault survivors’ experiences of reporting and the criminal justice system. The research was entirely exploratory, allowing the researcher to learn about the participants’ experiences.

Thematic Analysis involves firstly getting close to the data and becoming familiar with the interviews. Following this, ‘in vivo’ codes were developed. Codes were then analyzed to look at any similarities, patterns, or differences between the codes. After working closely with these codes, concepts were created and related to each other. Main themes as well as sub-themes were then generated, and a thematic map was used to explain how the themes and sub-themes fit together. A theme table was created, and the themes were defined. Throughout this process of thematic analysis, a reflexive diary was used to record any thoughts the researcher had. Finally, the key themes and sub-themes were discussed in the results section of this report.

Triangulation processes were implemented to increase confidence in the data. The last author conducted a ‘light-touch’ thematic analysis using the same processes as the first author. At each step, the two analyses were compared, and any key differences were noted and recorded in a separate reflexive diary. These differences did not adjust the overall theme nor theme structure, but some of the descriptions were modified following a prolonged reflection. This was further refined during the peer-review process.

### 2.4. Ethical Considerations

The nature of this research was highly sensitive and personal, requiring participants to revisit their experiences of reporting a sexual assault or rape. Several considerations and adjustments have been put in place to minimize any distress to the participants. This study received ethical approval from Bournemouth University’s Research Ethics Committee.

The interview criteria were set to ensure that we only explored participants’ experiences of reporting sexual violence or rape to the police and their experiences of the criminal justice system. We did not ask any questions relating to the assault. The interviewer reminded participants not to disclose or recount their specific sexual trauma information about the assault for their own emotional safety, and was trained in how to shift the focus of the interview should participants attempt to discuss their assault. Further clear boundaries were set for participants at the start of the interviews. The interview questions were also devised with assistance from those with lived experience at STARS Dorset to ensure that they focused on areas that are relevant to survivors and counsellors and that they would not be triggering. While these methods were introduced to reduce the potential impact for participants, we were keenly aware that survivors’ experiences of the criminal justice system can be considered as a ‘second rape’ (Campbell et al., 2001) and can add to retraumatization. Thus, additional safeguards were put in place for this work.

Participants were only invited to participate in this study after being screened by the Clinical Lead for STARS Dorset. This screening was designed to ensure they were not likely to be harmed, nor that their recovery would be impeded by partaking in the interview. Before signing the consent form, participants were given a detailed information sheet confirming what the study involves.

Interviews were held in an environment where participants would feel most safe and comfortable. The Clinical Lead for STARS Dorset was present during every interview, providing any emotional support where necessary and appropriately debriefing all participants. The debrief included the contact details of the researcher, the research supervisor, the clinical lead at STARS Dorset, the STARS Dorset helpline and Samaritans 24/7 helpline for urgent support.

The interviewer received extensive ethical training and specific training on the key areas surrounding sexual assault and rape. This included sensitivity training and active listening, in addition to confidentiality and protection of participants. The interviewer also received psychological debriefing support after each interview.

## 3. Results

**Figure 1:**
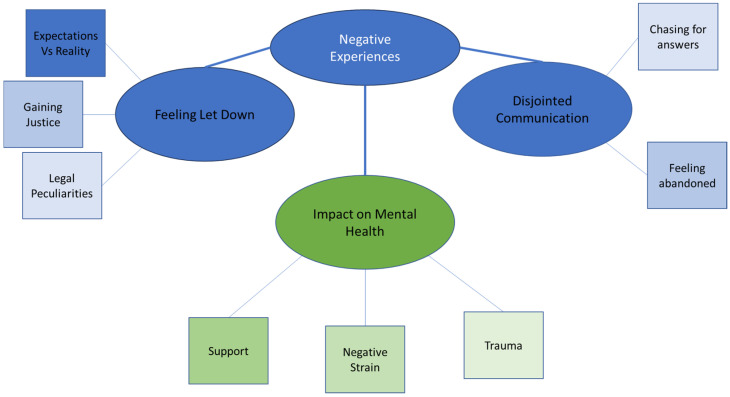
Thematic map showing the superordinate theme broken down into the three main themes and nine subthemes.

From the thematic analysis, one superordinate theme emerged, with two main themes focusing on the practical experiences of the survivors that led to a third main theme focusing on the psychological impact of their experience. Each of the main themes had sub-themes. While the focus of the work was on the psychological impact of going through the criminal justice system, it was important to honour the participants’ discussions and experiences. Figure 1 shows the theme map.

It was clear during the interviews that our participants valued the opportunity to discuss the many negative experiences that they had faced during their progress through the criminal justice system. These negative experiences were based around the disjointed communication they had received, leading the survivors to feel let down and the detrimental impact on their mental health.

### 3.1. Feeling Let Down

The first theme of “feeling let down” refers to the ways in which survivors reported being dissatisfied with certain aspects of going through the criminal justice system. This included survivors’ expectations falling short of the reality of reporting sexual assault or rape to the police and getting a conviction (sub-theme “expectation vs. reality”). It also involved whether survivors felt they got the justice they deserved from going through the criminal justice system (sub-theme “gaining justice”). Finally, survivors discussed the peculiarities of court proceedings and how they feel let down by them (sub-theme “legal peculiarities”).

#### 3.1.1. Expectations vs. Reality

Throughout the interviews, survivors reflected on how their expectations of reporting and going through the criminal justice system compared to the reality of it. Some of the differences between expectation and reality were positive, especially with some of the support services. Largely, however, survivors reported disappointment and anger with the handling of their cases, with descriptions of disorganization, slow, and unsupportiveness. Jessica explained how “It was more isolated than I ever thought it would have been.” Feeling let down with the reality of the process is also shown by Sophie, explaining that it “all sounded sort of fairly quick. It won’t take a very long time and I’ll keep you updated all the time. And really, that wasn’t the case.” Similarly, Claire “wrongly assumed that the police would look into everything, every corner, every avenue. Boy, was I wrong? Massively wrong.” Susan explains how she is “just absolutely furious with the way it’s been handled” in part because of her prior experiences with the police. Part of the reason why the survivors felt that their expectations did not meet reality was because of their intent or hope to gain justice and the difficulties in obtaining it.

#### 3.1.2. Gaining Justice

Gaining justice for the trauma survivors had experienced was the primary motivation they went through the criminal justice system in our study. ‘Not guilty’ verdicts were indicated as being a point of being let down and a failure to gain justice. Claire mentions how “The man in question was found not guilty. I have a lot of grief about [it] because I feel I was extremely let down in court.” Claire also explains how “when you’re there, you believe that justice is going to get done. And in my case, it certainly didn’t.” Laura describes the difficult process she has to go through in order to try and gain justice, which she felt was all for nothing when she says “I had to go to a specialist, specially trained officer to talk about the incident itself, to talk about really personal things, about my sex life and, you know, things like that. So, you know, and that was all videoed, and that was a really difficult experience. And I kind of felt that was all for nothing.” The sub-theme of gaining justice also relates to feeling believed. Survivors felt that being believed by those in the criminal justice system was the first step to gaining justice. Rebecca explains this by saying she feels “there needs to be something more to be able to allow people, victims, men or women who are subjected to go into court and feel reassured that they’re going to be believed.” This also links to Chloe’s experience of trying to get her case to court, explaining, “I do feel finally, two years later, we’re being taken seriously and I am doing the right thing.”

#### 3.1.3. Legal Peculiarities

Another common topic mentioned in the interviews was how survivors feel let down by the way they were treated throughout their time in court. This impacted how they may have or may not have obtained justice. Survivors reported they felt betrayed because the evidence they had provided was not read or presented to the courts, which they felt would be relevant. Furthermore, the feelings of being abandoned during a case disturbed some of the survivors. For example, Emma explained that “meetings should be not taking place when court is in session… I freaked because I was going to be sat there on my own with no-one.” Additionally, Sophie discussed how “they didn’t read all of my personal victim statement” and that she “found that really, really upsetting.” Claire shared her belief that court is “all about the theatre. They love it. The criminal justice system loves the theatre of it. They’re not interested in getting the right result. That is my opinion of the CPS.”

### 3.2. Disjointed Communication

The second main theme of disjointed communication represents how survivors report different experiences of their communication with the police and the Crown Prosecution Service (CPS). Some parts of the disillusionment with the criminal justice system, including feelings of being let down, may have resulted from the disjointed communication received by survivors as they went through the criminal justice system. While some survivors report having a particularly negative experience of working with the police and/or CPS in their hope to gain justice, other survivors discussed positive interactions with the police and/or CPS. Nonetheless, the positives and negatives stem from similar concepts around timeliness (sub-theme “timeframe of communication”), feeling abandoned, leading to feelings of powerlessness (sub-theme “abandonment leading to powerlessness”).

#### 3.2.1. Timeframe of Communication

Frequently, survivors reported issues with the timeliness of communication between themselves and the CPS or the police. When calling the non-emergency police number to report the sexual assault, Susan said how “it was half an hour before somebody answered and I was holding the phone thinking. If somebody doesn’t answer, in a minute, I’m going to give up.” Sophie explained that “It could be weeks and weeks before you hear anything.” Similarly, Chloe explained how she had “not even a phone call [from the police], to say actually, we’re a bit busy, but we will sort something out.” Furthermore, Jessica also experienced a lack of communication with the police as she “probably got about two calls in the space of three months.” When it came to communication with the CPS, similar experiences were discussed. Claire explained how the CPS “feel sort of untouchable. And they’re making all the decisions, and they have control over your life” because she was unable to communicate with them.

While there were a lot of negative comments made about the disjointed communication and the resulting feelings of abandonment and powerlessness, several of the survivors reported positively about the support they received. The comments made by survivors mentioned being helped and supported because of being communicated with in a timely fashion and being met on time, suggesting that feelings of support stem from feeling valued and respected. Laura explains “I can’t really fault them with how they dealt with it, and they did keep me updated all the way along as to what was happening.” Equally, Chloe explained “I haven’t had conversations er contact with [CPS] as such, and they’ve just had to do their bit er for the police [yeah], but they said it takes six weeks and they got back to us before that. So they’ve been brilliant.” Jessica felt she was given regular updates through the caseworker she was provided with through the CPS. She explains that “the courts were good. I got given a caseworker who was actually so helpful, more helpful than the police, and she was always on time giving me updates like helping me write statements out.” The emphatic positivity shown when describing these aspects of support contrasts completely with the dourer descriptions of the lack of support.

#### 3.2.2. Abandonment Leading to Powerlessness

Nuanced in the preceding discussion is the feeling that survivors had of being abandoned (and indeed let down) by the poor communication they received. Communication impacts how people feel and their self-esteem. In this way, leaving survivors to initiate contact with the police would have a negative impact on their wellbeing (Hu et al., 2025). Survivors felt they had to contact the police to get an update, making them feel their case and experience were not important enough. The words “chase” and “chasing” were used by survivors when asked about their experiences of communication. Chloe explained, “I was e-mailing and already felt like I was just being a pain.” Similar to this, Susan “had to keep chasing it every couple of months, to say, you know, what’s happening is there any update?” Jessica explains how she “had to write down all the questions for the next phone call. So any questions I actually had. For either the next phone call or the next visit that I had, which was quite frustrating because a lot of that I could have learnt by myself, but I just didn’t understand.” This point from Jessica highlighted how, if she had been provided with adequate information, she would have been able to help herself more. Instead, she had to wait for the state representative to make contact to further her knowledge. Lastly, Laura was surprised not to have received the support she was told to expect (including allocation of appropriate support workers), and it wasn’t until she followed this up herself that she was given this support. This is shown when she explains to the police, “I haven’t had this barrage of support what’s going on. And [police] had to make some phone calls and make it happen. So I did have to chase it.” All of the above examples highlight how individual failures within the criminal justice system can contribute to negative feelings and emotional distress of survivors of sexual violence. Indeed, Susan put it “you get given the card with the number on your phone the number nobody answers, it just trails away and you email, you don’t get a response and you’re just sat thinking, why do I contact people?”

While a number of survivors reported understanding that the police and CPS were over-worked and over-stretched, the consequences of this also increased the sense of abandonment in the survivors. Even if there is nominal support available, with the staff being overstretched, it creates a scenario where the survivors feel let down by the whole system, and this can include society as a whole. Claire commented that the officer she dealt with was a “Nice enough chap, but obviously doesn’t have enough time to deal with all my questions.” The resulting feelings of powerlessness were summarized by Rebecca thus, “they just came in and were very powering over me sort of thing and very intimidating and quite scary, because actually your life, you feel your life just completely got in the control of someone else’s hands.” Indeed, for Sophie, the whole point of going through the criminal justice system was for “not wanting to be silenced anymore and taking back control of my life.”

### 3.3. Impact on Mental Health

It is clear from the preceding pragmatic descriptions provided above that there are significant emotional impacts of every aspect of the criminal justice system on the wellbeing of survivors, and the final theme encapsulates this. Even small acts have repercussions on feelings of wellbeing, abandonment, isolation, helplessness, and powerlessness. It is no wonder that every survivor interviewed in this study reported that their experiences of the criminal justice system had an impact on their mental health. This is in addition to the direct trauma that they have experienced from their assault. Mental health has also been impacted by the distressing process of reporting the sexual assault or rape to the police and going through the criminal justice system. Survivors’ mental health has also been impacted by support from various individuals, including charities, the police, and external contacts such as therapists. The following sub-themes were developed from the interview transcripts: “support”, “negative strain” and “trauma”.

#### 3.3.1. Support

Firstly, the positive impact of having support when going through the criminal justice system for sexual assault or rape is demonstrated through examples provided by survivors. Claire explained how the police “recommended STARS, which was amazing. And you know, they have been my absolute saving grace. So I was really lucky to find them.” Furthermore, Emma says that “Without having [therapist] I wouldn’t have gone through with the case.” Conversely, the lack of support was detrimental to the emotional wellbeing of the survivors. Claire discussed how she felt she had a lack of support after going to her GP. She explains, “I’d been down the avenue of the GP and there was no support out there for me. They even wrote back to me and said I was too complex. To be dealt with. Really helpful. Yeah, you know.” When asked how this impacted her, Claire said she felt “Suicidal” from this lack of support.

#### 3.3.2. Negative Strain

A common area discussed by survivors was how their mental health was negatively impacted through having to go through such a difficult process of reporting and going through the criminal justice system. Claire explained that “there’s been lots of times where I’ve been like, I’m going to just drop it. It’s not, it’s not worth it at all.” Likewise, Sophie discussed how “It’s the hardest thing I’ve ever had to do, and I was terrified going in, waiting to go in. And all the months running up to it.” This was referring to the months of waiting before the court case. The prolonged delay, knowing that they would have to relive the assault again and face their assailant, produced an ongoing strain on the survivors, with Sophie “feeling like absolutely having a meltdown” as a result. The fear of not achieving their aim of obtaining justice by getting a guilty verdict weighed heavily on the survivors. Claire was brought to tears at the thought of having another not guilty verdict, explaining that she was “worried, very, very anxious and worried. I can’t have another not guilty verdict [cries]. Sorry I was near the edge of it.” Finally, Laura also reports the negative impact of having a guilty verdict when she explains that “there wasn’t enough evidence so that I remember that day really vividly and how upset I was.” Then, at the end of it, the criminal justice system leaves the survivors “to pick up the pieces of everything you’ve just been told” without any support, further adding to the abandonment feelings.

#### 3.3.3. Trauma

Throughout the interviews, it was clear to see the impact that the trauma they have experienced going through the criminal justice system has had on their mental health. Remembering that the interviews focused on the experience of going through the criminal justice system, rather than the assault, the comments below highlight how traumatic obtaining justice can feel. This was especially difficult as the survivors were made to relive their trauma as part of the process of reporting and going through the criminal justice system. The trauma of going through the criminal justice system occurs against the backdrop of the original assault, and the survivors found it difficult to separate the two sets of trauma. Laura explains, “I’m still left with, you know, emotional difficulties from what happened. And you know, it’s it leaves a very deep scar.” Furthermore, Emma describes the huge impact the trauma has had on her life when she talks about “the years of my life, it’s destroyed not being able to work because of that. And. I think the fact that it has left me with such severe mental health for all these years.” The years of impact were increased by the timescale of her journey through the criminal justice system. Rebecca also reports getting physically unwell as a result of not being able to sleep when she says, “I made myself quite ill, I wasn’t sleeping and I was petrified every night that someone was out to get me.” Emma points out the effects of the trauma are long-lasting: “I can go 12 months and be absolutely fine, and then all of a sudden bang nightmares flashbacks.” Lastly, to summarize the huge impact of the trauma on a survivor’s mental health, Jessica explains that “next to nobody would go to the actual police and report if it isn’t true.”

## 4. Discussion

In this study, we explored the experiences of sexual assault survivors who have gone through, at least part of, the criminal justice system. The aim was to expand on previous work highlighting the pragmatic difficulties faced by survivors within the criminal justice system (Champion et al., 2021) to explore the psychological consequences of those difficulties. Consistent with Champion et al., we found that survivors have struggled at various parts of the process (difficulties with the timeliness of communication, procedural confusion). The psychological consequence of these struggles is that survivors feel let down by the criminal justice system. The route of this stems from the juxtaposition of the desire and difficulties in obtaining justice.

Being believed and obtaining justice were key elements for a survivor reporting and going through the criminal justice system (Konradi & Burger, 2000). Wheatcroft et al. (2009) have shown the negative impact of not being believed when reporting sexual assault to the police, including revictimization. Slow and disjointed responsiveness from the criminal justice system led to the survivors in our study feeling abandoned and isolated. The comment made by Claire highlights how a survivor of sexual violence can feel powerless to get justice and, in their lives, generally, even long after the assault, when they are trying to push for change. Such a feeling of helplessness can contribute to depression and anxiety (Pryce et al., 2011). Indeed, the sense of powerlessness because of the authority of the police and CPS impacted our survivors’ long-term mental health. All of this contributed to negative strain on the survivors’ wellbeing and additional trauma of the criminal justice system. Such results are entirely consistent with the notion that poor investigative practice leads to survivors feeling they are of low priority, leading to damaged feelings of self-worth and dignity (Jordan, 2022). Given that survivors may feel that the members of the criminal justice system are representatives of the state (Sunshine & Tyler, 2003), a lack of well-timed, helpful communication can exacerbate feelings that the state does not care about the individual nor the crime (McGlynn & Westmarland, 2019).

As shown in the results, participants reported suffering with their mental health in various ways. This impact on mental health was also affected by the support networks surrounding the survivor. Claire’s deep worry and anxiety highlighted the emotional impact of the court process and trying to gain justice. Such emotional impact led others to illness. This shows the lasting effect of the trauma experienced during the assault and the retraumatization through the criminal justice system, consistent with findings published in the literature (Sanderson, 2009; Campbell & Raja, 2005). Disorders such as anxiety, depression, and post-traumatic distress were all common for individuals who have experienced sexual trauma (Campbell et al., 2001). Our findings add to this by highlighting how going through the processes of the criminal justice system adds further trauma and exacerbates symptoms (McFarland et al., 2019). Indeed, the process of journeying through the criminal justice system is additional trauma to the survivor (Hohl et al., 2025).

The findings of the current study expand on research by Park (2015), who researched the essence of the survivors’ experiences of reporting sexual assault in the US. There were several consistent themes emerging across the studies in this area: the mixed response from the criminal justice system, the poor communication from the criminal justice system and its negative consequences. Where our study deviates from the results of Park’s work is in the motivation behind why the survivors chose to report the sexual assault. Park found that the reasons for reporting were to improve the safety of others, improve the data on sexual violence, and alleviate their own fears, while we found that gaining justice was the primary motivation. Given that the sample size in both studies was appropriate for qualitative methods, it highlights a potential limitation in the generalization of the findings. It also might highlight cross-cultural differences in the motivation to report and beliefs about law enforcement.

There are significant practical implications of the current work, enhanced because of the replication and extension of the work of Champion et al. (2021). Champion et al. have provided a list of excellent recommendations for improving the experience of survivors through the criminal justice system, and these will not be repeated here. Indeed, the CPS has taken on many of the recommendations for improving communication with survivors of sexual violence, including regular face-to-face meetings and clear letters detailing decisions (CPS, 2025). It is established that agency, prevention, and connectedness are key drivers in conceptualizations of justice for survivors of sexual violence (McGlynn & Westmarland, 2019), and the advances in process may help with this. Further emphasis, however, will be placed on two aspects here that further increase agency and connectedness for survivors. One: providing suitable information to survivors about the criminal justice process and two: providing emotional support to survivors of sexual violence.

Survivors need to be aware of the practicalities of going through the criminal justice system. This procedural information about charging decisions, timeframes, who to contact, and the legal peculiarities is vital for empowering survivors. The antiquated legal structure thus does not regard survivors as people, but rather as a commodity of evidence to be used to get a conviction. This point highlights how a lack of empathy toward the survivors of sexual violence is inherent within the way UK law treats survivors as witnesses and may add an avenue of training to those within the criminal justice system (the police and the CPS). Such additional supportive information would help allow survivors to have control over their case, enable agency over how to source information, and potentially therapeutic jurisprudence (Wemmers, 2013).

Secondly, the support needed for survivors of sexual violence needs to be tailored to the fact that the journey through the criminal justice system is long and arduous. It prevents full recovery until it is complete, necessitating the need for specialist trauma-informed counselling services to be available to survivors. Currently, in the UK, these are provided by charities or via private clinicians. Given the significant emotional and financial impact of sexual violence on survivors, this seems like an oversight warranting further investment. Nonetheless, the emotional support that survivors need, based on our sample, is to ensure that they do not feel isolated, abandoned, and powerless. Rather, it should be about providing a source of support and strength from which the survivor can grow and find their recovery.

A further implication of the study, already implied, is that there is significant variability of experiences of survivors within the criminal justice system, even within the same Police Force. The survivors in our study reported conflicting experiences of communication from members of the criminal justice system. Chloe explained how she had “not even a phone call, to say actually, we’re a bit busy, but we will sort something out” showing how the police lacked satisfactory communication when dealing with her case. These findings are consistent with previous research suggesting that treatment of survivors by police can be poor (Campbell & Raja, 2005). Other survivors reported that the police officers and CPS lawyers were excellent in their manner. This indicates that either police officers have different levels of training in dealing with sexual violence or that other factors (such as individual focus, personality, and work ethic) play a role in how those officers deal with survivors of sexual violence.

There is specialist training for police officers in the UK for interacting with survivors of sexual violence. Not all officers will undertake this, which explains why both Champion et al. (2021) and ourselves found different experiences with police officers. Furthermore, different attitudes among the police officers might also explain the different experiences that survivors experienced. Venema (2016) reported how some police officers have distinct schemas that are related to rape myths and stereotypical views. Rape myths are stereotypical and false views about rape, sexual assault, perpetrators, and survivors (Lonsway & Fitzgerald, 1994) that can lead to different responses to and treatment of survivors, affecting attrition and negative outcomes for survivors (Grubb & Turner, 2012). This indicates a need for clinical support or supervision in the police to adequately tackle such issues.

The qualitative interviews conducted in this study allowed the researchers to understand the meaning behind the experiences of survivors of sexual assault. Participants were able to openly discuss their personal experiences, allowing for more in-depth data collection, which was only possible by using a qualitative data collection method. Thematic analysis enabled the researcher to take a flexible approach to data analysis, and the participants’ own words are used throughout the research to retain the authenticity of the survivors’ voices.

Despite the benefits of this qualitative research method, our epistemological position within social constructivism highlights the need to acknowledge potential bias, as the researcher’s preconceptions, previous understandings, and knowledge may have influenced the analysis. Steps were taken to minimize these risks, such as multiple researchers within the research team analyzing the data to ensure results stay true to the data, such as during the initial coding stage of thematic analysis. Of course, the findings from this study cannot be generalized to every individual who has reported sexual assault to the police and gone through the criminal justice system, not least because all of the participants in the current study were female. Given that, we believe that knowledge is socially constructed and subjective, and different people will experience and interpret events according to their experience and beliefs. Gender-specific barriers to reporting sexual violence exist for men (Widanaralalage et al., 2022) and may operate in addition to or in concert with the barriers faced by women, especially considering the social norms around masculinity (Kong, 2019). Nonetheless, this study does provide valuable insight into areas in which there are inconsistencies in how survivors are treated.

## 5. Conclusions

The current findings confirm the psychological impact of sexual assault and having to report it to the police and go through the criminal justice system. This includes trauma and negative strain from court and support, which all have an impact on the survivor’s mental health. Additionally, the incongruent nature of communication between the survivor and the police and criminal justice system highlights an area for improvement, as it is not yet adequate for every individual. This suggests various avenues in which a survivor’s experience with the police and the criminal justice system can be improved to make it more comfortable for the survivor and give them the best possible chance at gaining justice.

## Data Availability

Due to the sensitive nature of the study, which involved interviews with survivors of sexual violence, the raw interview transcripts cannot be publicly shared, as they may contain information that could affect future legal proceedings. However, the thematic analysis table is available online and has been provided to the reviewers. Redacted versions of the interview transcripts may be made available upon reasonable request.

## Notes

1 Scotland has a subtly different legal system from England and Wales, including verdicts at the time that our participants went through the criminal justice system.

2 In UK law, someone who reports a sexual assault is not considered a victim until a guilty verdict has been passed. Until this point, they are legally considered a witness. Because this work is based on the psychological effects, we have chosen to use the phrase “survivor” throughout.

3 [Charity name] is a charity that provides support and counselling for all survivors of sexual violence living, working, or studying in County, UK.
